# Volume-outcome relationship in critical care: a systematic review

**DOI:** 10.1186/cc11128

**Published:** 2012-03-20

**Authors:** DJ Wallace, YL Nguyen, L Trinquart, DC Angus, P Ravaud, JM Kahn

**Affiliations:** 1 CRISMA Center, University of Pittsburgh School of Medicine, Pittsburgh, PA, USA; 2 Centre d'épidémiologie clinique, CHU Hôtel Dieu, Paris, France

## Introduction

The relationship between provider volume and patient outcome has been demonstrated for many medical and surgical services, including critical care. This relationship is used as one rationale for regionalization of adult intensive care. However, the volume-outcome relationship is not always consistent across studies, and it has not been explicitly evaluated in a heterogeneous population. We performed a systematic review of studies that assessed the association between volume and outcome among critically ill adult patients.

## Methods

We searched the MEDLINE and EMBASE databases for articles published between January 2001 and December 2011 using medical subject heading terms and text words for conditions related to critical illness in adults. Trauma studies were excluded. Two study investigators independently reviewed titles, abstracts and articles identified from the search algorithm and abstracted study-specific data using a standardized abstraction form. Variables of interest included study characteristics, patient characteristics, study period, volume definition, primary and secondary outcomes, risk-adjustment methodology, statistical analyses, results, risk of bias and funding body.

## Results

We reviewed 80 studies, of which 27 (34%) met all inclusion criteria. Studies were excluded most commonly when the majority of the patients did not require critical care (*n *= 46), the study was presented only in abstract form (*n *= 4), data were duplicative (*n *= 2) or an outcome measure was not assessed (*n *= 1). One publication included three different patient populations; these were counted as separate studies. The final 29 studies represented seven clinical categories: respiratory (*n *= 9), postoperative (*n *= 7), cardiovascular (*n *= 4), general admissions (*n *= 3), sepsis (*n *= 2), neurological (*n *= 2) and gastrointestinal (*n *= 2). Eighteen studies (62%) demonstrated a statistically significant association between higher patient volume and better health outcomes, although the magnitude of the relationship varied across diagnoses. No study showed a statistically significant association between higher volume and poorer outcomes.

## Conclusion

The majority of studies evaluating the volume-outcome relationship in critically ill patients demonstrated better outcomes with higher clinical volumes. There was variability in the association across diagnostic categories, indicating that quality improvement efforts based on the volume-outcome relationship such as regionalization of care may be more successful in specific patient subsets.

